# 

**Published:** 1888-01-21

**Authors:** 


### NOTICES.

The scale of charges for small prepaid Advertisements—Situations Wanted, Situations Vacant, &c., &c., is as follows:—

| | s. | d. |
|---|---|---|
| One insertion of three lines | 1 | 0 |
| Three „ „ „ | 2 | 6 |
| Per line afterwards | 0 | 6 |

*A line averages ten words.*

All copy for Advertisements must be sent to A. P. Watt, 2, Paternoster Square, E.C., by first post on the Tuesday *preceding* publication.

**All communications respecting editorial matters** should be addressed to the Editor, 38, Old Jewry, Cheapside, E.C.

### QUESTIONS AND ANSWERS.

A READER OF "THE HOSPITAL," CHARD.—We never give advice or recommend particular medical men.—ED. *T. H.*

### AMUSEMENTS WITH PROFIT FOR ALL AGES.

**Rules.**—All answers must be addressed to the PRIZE EDITOR, THE HOSPITAL, 38, Old Jewry, Cheapside, London, E.C., not later than the *first* post on Thursday next. The full name and address must in all cases be given, but a *nom de plume* may be added for publication. Only one side of the paper must be written on.

### FOR MEN AND WOMEN.

### Acrostic Competition.

A PRIZE OF ONE GUINEA will be given to the competitor who gains the highest number of marks for best original acrostics during the ensuing quarter. All acrostics sent in for this competition will be credited according to merit, twelve marks being the highest score given for one acrostic.

A PRIZE of HALF-A-GUINEA to be given to the competitor who gains the highest score for solution of acrostics set, including the one inserted on November 26th. One mark only will be given to the competitors who solve all the lights of each acrostic.

These competitions to alternate weekly.

This week credit will be given for solution of the following :—

No. 5. DOUBLE ACROSTIC.

To write my *second* is no easy task,
That it should be my *first* is much to ask.

Once the delight and thing " to do " of town,
Alas ! my palmy days are going down.

My old-world strains no longer welcome sound,
So in " collections " I alone am found.

Complete in every part am I,
A whole in all entirety.

In Santa Croce lies his honoured dust,
Who told the sky's great history to the unjust.

" Fleet-footed messenger " of many hues,
Thee for a " light " I take to help my muse.

Alone I'm nothing, yet I count a score
If but a magic two be placed before.

To prove me is the prisoner's last resource,
And if successful oft impedes Law's course.

To chop me is the work of scholars now,
But they must spend some pains in learning how

No. 4. DOUBLE ACROSTIC.

**Answers.**

| | | |
|---|---|---|
| D ea F | | "Goldsmith." |
| E mbry O | | " Shenstone." |
| S cimita R | | " Siege of Corinth." |
| T ren T | | "Marmion." |
| I o U | | " Merchant of Venice." |
| N elso N | | "Tennyson." |
| Y ul E | | "Marmion." |

Correct answers have been received from Reldas, Lady Clara. Ellice.—You should have been credited with 12 marks last week for " Original Acrostic."

### II. Word and Literary Competition.

Commenced November 5th, 1887 ; ends January 28th, 1888.

Three prizes of one guinea, 15*s*. and 10*s*. 6*d*. will be given each quarter to the three competitors who obtain the highest number of marks : (1) by making the largest number of words derived from the word or phrase set for anatomical dissection ; or (2) for the best answers to (6); or (3) by (1) and (2) combined.

(A) THE WORD COMPETITION.

We shall give the newspapers for dissection during this quarter, that for this week being

" THE FIELD."

OBSERVE.—Proper names, abbreviations, foreign words, words of less than four letters and repetitions are barred. Nuttall's *Standard* dictionary only to be used.

### Results of Tenth Week.

*Daily News.*—Ellice (236), Gretta (209), K.M. (143), Condorcet (142), Reldas (141), Lauriston (130), Kath (129), Brisbane (127), E. E. (85).

### Letters from Across the Sea.

SMILAS sends the following from Texas :

I could not write to you from San Antonio, my hands being very sore from what is called " prickly heat " ; but I found your letter there saying you enclosed five dollars, but there was not any money in it ! When we got to New Orleans, we did not proceed by train as I thought we would, but by boat up the Missisippi to Thilbideaux, and from there to San Antonio, where we were for three days before I got anything to do. I am now at work on a farm about two days' ride from San Antonio, and although such work is new to me I can manage it fairly. There is no post office nearer than a day's ride from this farm, and I can only get letters when some of us have time to ride over there on business. F—— and his family are on a farm about four miles from here. The people I am with are Irish, and have been here about seven years. I have 12 dollars a-month, board, lodging, and washing, and free grazing on the farm if I like to buy a horse (when I have the money). This I may do when I get my wages. I have 21 cows to milk every morning, 15 pigs to feed, and seven horses to take to the water. I have had young F—— here helping me for the last four days. It is very hot, and we cannot work during the middle of the day. The mosquitos are very bad here, yet, strange to say, they will not touch me. As I am the only man on this ranche, I cannot go to the post-office, but F— is going there this afternoon, and will post this and bring out any letters there may be ; but as he goes to his own place I may not see him for three or four days, so you can judge how very difficult it will be for me to get any news from anyone. I hope all are well at home. I cannot write well yet, as my fingers are as stiff as wood, and only able to achieve downstrokes. Although the work is real hard manual labour, I feel well and as strong as a horse. The food and lodging is good. I am never tired, though I am up before sunrise, and rarely get to bed before ten o'clock, as I have to pen all the cattle and drive the calves out. During the day I cut down wood for firing, and load the wagon with all I can and drive it home. I have little time for writing, as during the hot part of the day I am away from the house, and cannot bring out paper, pens, and ink when leaving for my work in the morning. I am told by all the farmers round here that with the wages I have I ought to be a ranche-holder in a few years, if I invest wisely in stock and take care of what I buy. Th s I can do when they are driving horses by here to the market. Sometimes they will have come a couple of hundred miles and are done up, poor brute ! and unable to go further, when their owners will gladly sell them for from two to five dollars each. By picking out the most likely ones and grazing them for a few months, they are then fit for the San Antonio market, and will fetch from twenty to one hundred dollars. This is how my present " boss " got on. Of course, really good animals are rare ; but as this ranche is on the main road I stand a very good chance. Yet despite these prospects (?) I would not advise H. to come out here. It is a very hard life, and one is never clear. Water is so scarce that you can never have a bath, as the lakes are all dried up, and we have to hoard what we can in wells and tanks for the cattle. Up to the present we have had no rain, and everything is burnt up. On the whole I like the life well enough. There is any amount of game here, but I have no time to get it. Now I must finish, and shall write whenever I get a chance.

Extract of a letter sent by *Ellice* from Tauranga, Bay of Plenty, N.Z. :

" There is one incident worth relating *in voyage* and it happened as soon as I went on board. I found my first-class cabin calmly appropriated by another young fellow. When interrogated he was found to have a second-class ticket, and said he was a teetotaller, and belonged to the Young Men's Christian Association. I stayed ten days in Auckland, thought it a very pretty place, but could not get any work to do there, so came on to Tauranga, where I got on a survey party directly. Here I met some very good fellows, who had come out as cadets on farms, but found to their cost it was all a ' swindle.' After I had been at Tauranga about a week, the survey party I was attached to went up to the Hot Lakes : camped out all the time, working from eight in the morning till five in the evening, with an hour for food in the middle of the day. The work was not particularly hard. We contrived to have some fun in the shape of a cricket match, " The World " against " Colonials." Our party played for the World. I made 0 first innings, and not out 46 in the second. We also indulged in athletic sports, and " The World " won nearly all the events. Shortly after this, I had a very exciting adventure which very nearly cost me my life. A party of us went out for a fishing expedition. In some strange fashion the boat capsized. We were in the water quite half-an-hour before we got rescued by a Maori boat which happened to be sailing by and heard us shouting. I lost nearly all my money, my watch and chain, papers, &c., and coat. With regard to money making, everything is very dull out here. The railway everyone was talking about seems to have fallen through ; in fact, the colony and people seem in an impoverished state. If you think of coming out, I should not advise you to do so unless you have something definite to come to, or don't object to doing hard work—I mean, of course, rough work. More details about the Maoris in my next."

### THE CHILDREN'S CORNER.

PRIZES.

FOR MOST CORRECT ANSWERS TO PUZZLES.

This Commenced October 1st, 1887.
One Pound a Month.

FOR BEST LITTLE LETTERS,
Ten Shillings per Quarter.

A PRIZE OF THREE GUINEAS

to the Competitor who shall have sent in the largest number of correct or best answers during the twelve months.

### Puzzles—Third Week.

No. 9. GEOGRAPHICAL CHARADE.—Add what everybody likes to have to a term signifying of small importance, and it will make a native state of India.

```
      .
    . . .
  . . . . .
. . . . . . .
  . . . . .
    . . .
      .
```

No. 10. DIAMOND PUZZLE.—1. Half of no. 2. A weapon. 3. A city of Italy. 4. The capital of an English county. 5. A river in France. 6. A French adverb. 7. A letter of the alphabet.

No. 11. ENIGMA.—Though mostly small and always very sour, with joy and frolic pass I many an hour.

No. 12. BIOGRAPHICAL ANAGRAMS.—1. Trap a bone. 2. I met all, Will.

### Results of First Week Puzzles.

No. 1. TRANSPOSITIONS.—Words. Swords.

No. 2. BIOGRAPHICAL ACROSTIC.

C heltenha M
O uriqu E
R e X
T ancred I
E ri C
Z en O

No. 3. ENIGMA.—Almanack.

No. 4. ENGLISH TOWNS ENIGMATICALLY EXPRESSED.—1. New-bury=Newbury. 2. Wind-sor=Windsor. 3. Gains-borough=Gainsborough. 4. Stock-port=Stockport.

*All right*—A. G S. McCulloch, P.S., Plummy, Fluff, Druce, Happy Eliza, Scrooge, Judy, Tim, Gem, Toad, A.C.K. *Three right.*—Pepita, Jacko, Jumps. *Two right.*—Excelsior, Spider, Robinson.

**Notice to all Correspondents.**—N.B.—All letters referring to this page, which do not arrive at 38, Old Jewry, Cheapside, London, E.C. by the *first post on Thursdays*, and are not addressed PRIZE EDITOR, will in future be disqualified and disregarded.
No one will be permitted to win the Monthly or Quarterly Prizes twice in any one year, the annual prizes being offered especially to prevent this.

